# Microbial Community Analysis of Anaerobic Reactors Treating Soft Drink Wastewater

**DOI:** 10.1371/journal.pone.0119131

**Published:** 2015-03-06

**Authors:** Takashi Narihiro, Na-Kyung Kim, Ran Mei, Masaru K. Nobu, Wen-Tso Liu

**Affiliations:** 1 Department of Civil and Environmental Engineering, University of Illinois at Urbana-Champaign, 205 North Mathews Ave, Urbana, IL 61801, United States of America; 2 Bioproduction Research Institute, National Institute of Advanced Industrial Science and Technology (AIST), Central 6, Higashi, Tsukuba, Ibaraki 305-8566, Japan; Fudan University, CHINA

## Abstract

The anaerobic packed-bed (AP) and hybrid packed-bed (HP) reactors containing methanogenic microbial consortia were applied to treat synthetic soft drink wastewater, which contains polyethylene glycol (PEG) and fructose as the primary constituents. The AP and HP reactors achieved high COD removal efficiency (>95%) after 80 and 33 days of the operation, respectively, and operated stably over 2 years. 16S rRNA gene pyrotag analyses on a total of 25 biofilm samples generated 98,057 reads, which were clustered into 2,882 operational taxonomic units (OTUs). Both AP and HP communities were predominated by *Bacteroidetes*, *Chloroflexi*, *Firmicutes*, and candidate phylum KSB3 that may degrade organic compound in wastewater treatment processes. Other OTUs related to uncharacterized *Geobacter* and *Spirochaetes* clades and candidate phylum GN04 were also detected at high abundance; however, their relationship to wastewater treatment has remained unclear. In particular, KSB3, GN04, *Bacteroidetes*, and *Chloroflexi* are consistently associated with the organic loading rate (OLR) increase to 1.5 g COD/L-d. Interestingly, KSB3 and GN04 dramatically decrease in both reactors after further OLR increase to 2.0 g COD/L-d. These results indicate that OLR strongly influences microbial community composition. This suggests that specific uncultivated taxa may take central roles in COD removal from soft drink wastewater depending on OLR.

## Introduction

As the global consumption of soft drinks continues to grow, 687 billion liters in 2013, the global value reach 830 billion USD [1]. However, this incurs copious production (up to 2.0 trillion liters per year) and discharge of wastewater [2] containing high concentrations of sugar [3–5] and polyethylene glycol (PEG; HO[CH_2_CH_2_O]_*n*_H), a detergent for bottle washing and equipment rinsing [6]. As such, the wastewater stream is characterized by high organic content with the COD ranging from 1.2 to 8.0 g L^−1^ and BOD_5_ from 0.6 to 4.5 g L^−1^ [3], and required to be treated to reduce COD to prevent the occurrence of contamination in the natural environment. Previous studies report physicochemical treatment, including reverse osmosis [2], filtration [2, 7], ion-exchange [2, 7], and ozonation [8]; however, such approaches are relatively ineffective for removing soluble compounds (e.g., PEG and fructose) compared with biological methods [5, 9, 10]. While aerobic biological treatment systems have also been applied [11, 12], long hydraulic retention time (HRT), high aeration requirement, extensive land requirement, high sludge production, and poor biomass settling are significant drawbacks [13].

Anaerobic biological treatment is a promising alternative due to its high capacity to degrade concentrated and recalcitrant substrates [13, 14]. Several studies have successfully applied anaerobic bioprocesses to treat soft drink wastewater, including immobilized cell bioreactors [15, 16], up-flow anaerobic sludge blanket (UASB) reactors [13, 17], anaerobic filters [18], and up-flow anaerobic pack-bed reactors [19]. Although these reactors achieved satisfactory COD removal, none of these studies report the microorganisms that facilitate degradation of the wastewater organic compounds. Without understanding of the microbial community structure and ecology, development of strategies to maintain and improve treatment efficiency and stability can be difficult. In the present study, we developed anaerobic bioreactors treating synthetic soft-drink-production wastewater and investigated the temporal change in microbial community structure during the operation through 16S rRNA gene pyrosequencing. Specifically, we identify organisms potentially related to reactor operational conditions.

## Materials and Methods

### Reactor operation

**Fig 1:**
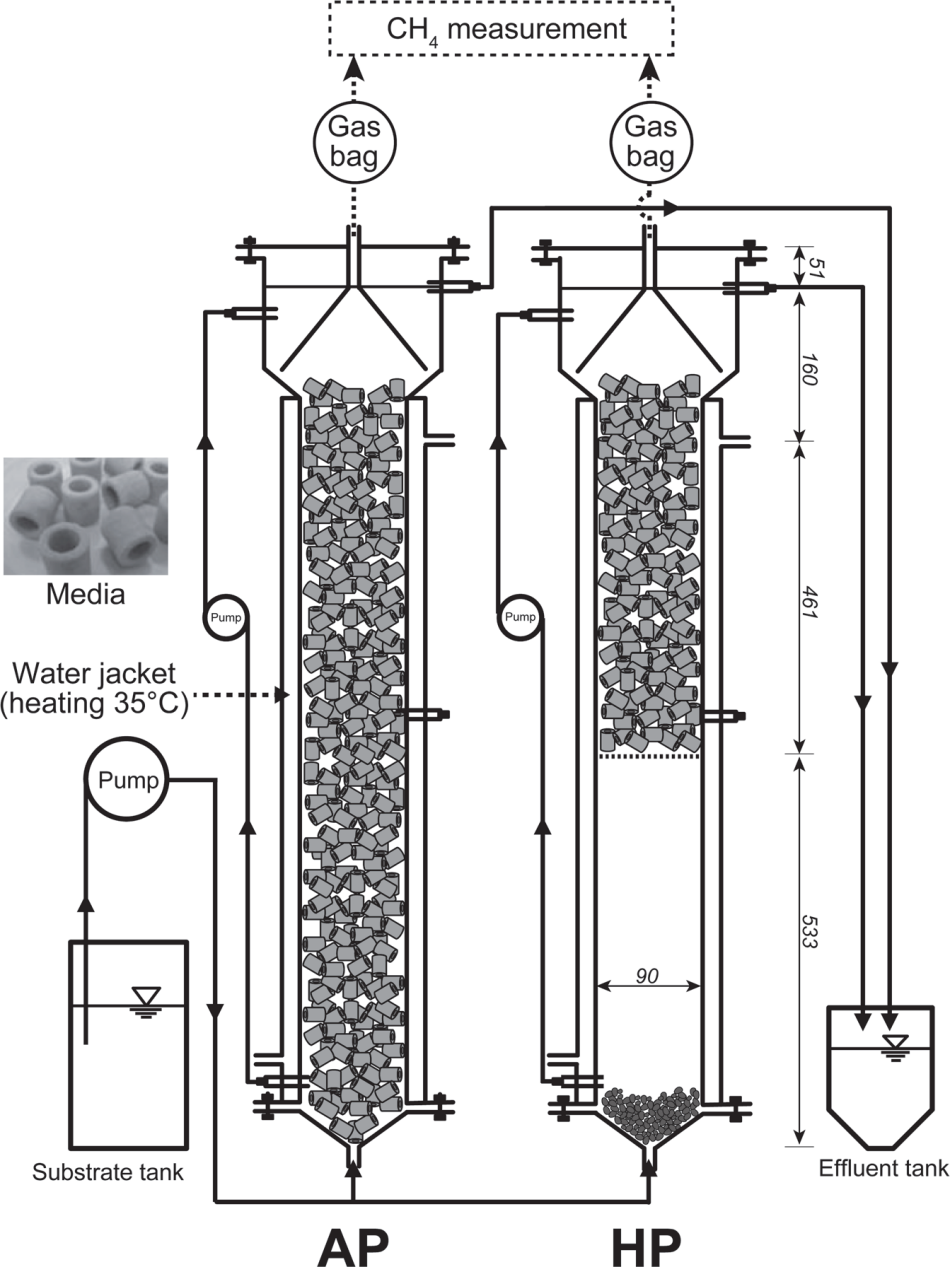
Cross-section illustration of the anaerobic packed-bed (AP) and hybrid packed-bed (HP) reactors. The reactors were equipped with water jacket and heated by water heater to kept at 35°C. The numbers in italics indicate size (mm).

**Fig 2:**
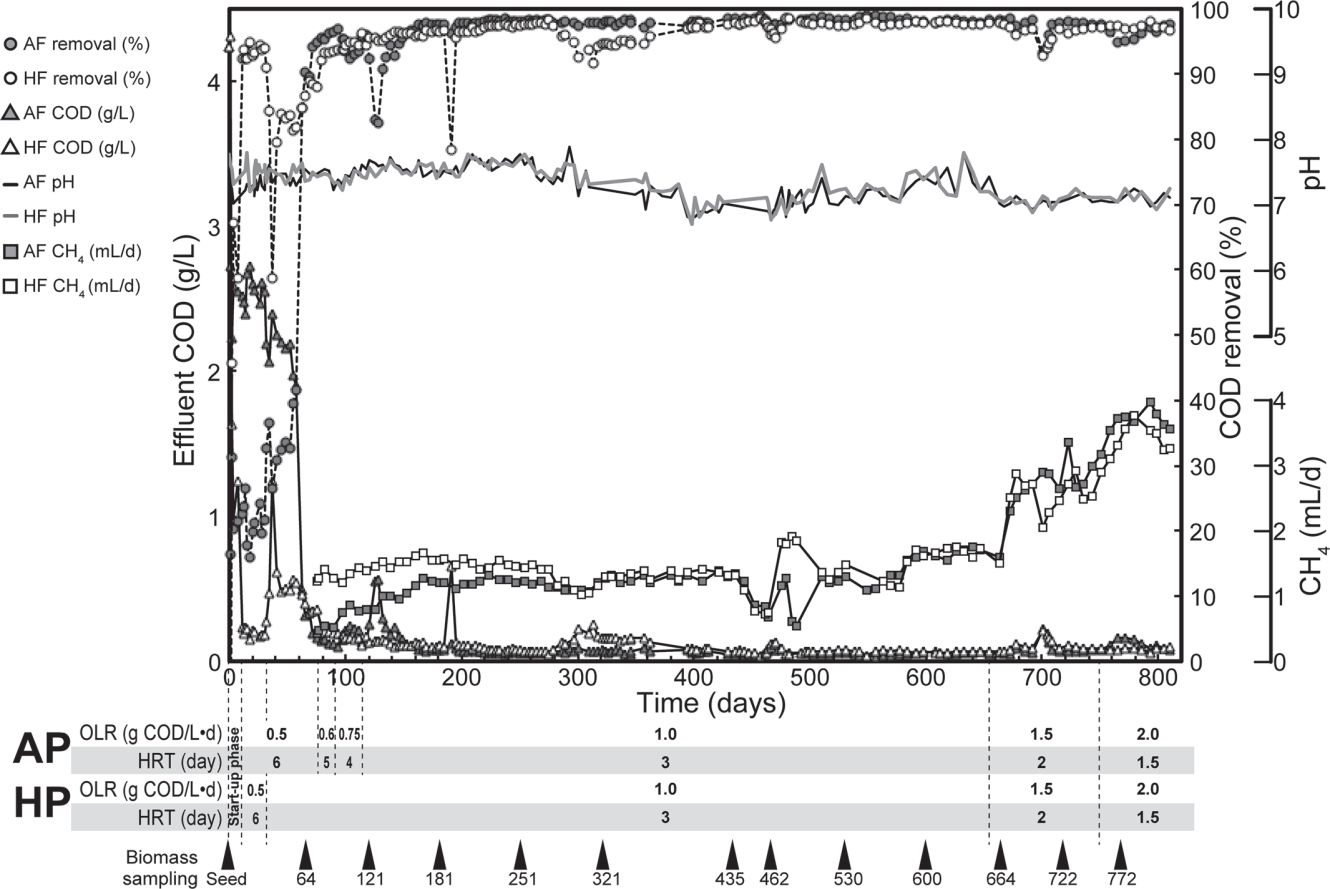
Changes in operational parameters of anaerobic packed-bed (AP) and hybrid packed-bed (HP) reactors. Closed circle, COD removal (%) in AP; open circle, COD removal (%) in HP; closed triangle, effluent COD concentration (g L^−1^) in AP; open triangle, effluent COD concentration (g L^−1^) in HP; black line, pH in AP; gray line, pH in HP; closed square, methane gas production (L d^−1^) in AP; open square, methane gas production (L d^−1^) in HP. The reactors were operated at different organic loading rates (OLR) ranging from 0.5 to 2.0 g-COD L^−1^ d^−1^. The COD concentration of influent synthetic wastewater was decreased due to absence of polyethylene glycol 200 (1,100 mg L^−1^) during days 398–411. The triangles in the bottom indicate the periods for biomass sampling from the reactors.

Two anaerobic up-flow bioreactors (7.6 L working volume) were operated separately at 35°C (Fig. 1). The anaerobic packed-bed reactor (AP) and hybrid packed-bed reactor (HP) were filled with the Siporax ceramic media (L×D×H; 15×15×15 mm) (Aquatic Eco Systems, Apopka, FL, USA) to fill 10.2% and 5.0% of their working volume, respectively. Seed sludge sample was taken from anaerobic digester at Urbana, IL, USA. The reactors were fed with 3,000 mg COD L^−1^ synthetic wastewater that mimicked the composition of wastewater discharged from soft drink-processing factory [3–5, 7, 15, 17, 20]: 1,100 mg L^−1^ of polyethylene glycol 200 (PEG200); 1,500 mg L^−1^ of Corn Sweet High Fructose 55 (ADM, IL, USA); 30 mg L^−1^ of acetone; 30 mg L^−1^ of ethanol; 10 mg L^−1^ of silicone grease; 16 mg L^−1^ of K_2_HPO_4_; 19 mg L^−1^ of FeSO_4_·7H_2_O; 366 mg L^−1^ of NaHCO_3_; 2 mg L^−1^ of NaF; 2.5 mg L^−1^ of NaOCl; and 28 mg L^−1^ of NH_4_HCO_3_. These components were dissolved in tap water, and pH was adjusted to 9.5–10.0 with 5 M KOH to maintain the pH at 7.3–7.8 in the AP and HP. The internal circulation rates were 300 mL min^−1^ for both reactors. The reactors were operated under different hydraulic residence time (HRT) and organic loading rates (OLR) ranging from 1.5 to 6 days and from 0.5 to 2.0 g L^−1^ d^−1^, respectively (Fig. 2). To avoid overloading of the organic compounds on initial microbial consortia, two reactors were operated for 11 days with constant recirculation of synthetic wastewater and no fresh influent. After day 11, both AP and HP reactors were fed with influent at a hydraulic residence time (HRT) of 6 days and an organic loading rate (OLR) of 0.5 g L^−1^ d^−1^. For AP reactor, the HRT was decreased to 5, 4, and 3 days and the OLR gradually increased to 0.6, 0.75, and 1.0 g L^−1^ d^−1^ at 77, 91, and 115 days of the operation, respectively. For HP reactor, the HRT was decreased to 3 days and the OLR increased to 1.0 g L^−1^ d^−1^ after 31 days. After day 655, the HRT was decreased to 2 days and the OLR increased to 1.5 g L^−1^ d^−1^ for both reactors. Furthermore, the HRT of both reactors was decreased to 1.5 days and the OLR increased to 2.0 g L^−1^ d^−1^ after 744 days of the operation.

### COD and methane gas measurements

The soluble COD was measured with COD digestion kit (HACH, Loveland, CO, USA) and DR/4000 U Spectrophotometer (HACH) according to the Standard Method 5220D [21]. Methane gas produced from the reactors was collected in gas sampling bag (Standard Tedlar PVF Bags, DuPont, DE, USA) and measured using a GC-2014 Gas Chromatograph (Shimadzu Scientific Instruments, Kyoto, Japan) equipped with a thermal conductivity detector (Shimadzu Scientific Instruments) and a Molecular Sieve 13X packed column (2,000×2 mm) (Restek, PA, USA).

### Biomass sampling

Biomass samples for microbial community analysis were collected from AP and HP at 64, 121, 181, 251, 321, 435, 462, 530, 600, 664, 722, and 772 days of the operation (Fig. 2). The ceramic media (ca. 5 pieces) were collected from 16 cm depth from effluent outlet with autoclaved forceps and put into 50-mL tube. After 10 mL of 1×PBS was added, the media was vortexed rigorously to remove the biofilm. After centrifugation (8,500 ×*g*, 3 min), the biomass samples were collected and stored in −80°C freezer until DNA extraction.

### DNA extraction, PCR, and pyrosequencing

DNA extraction, PCR, and pyrosequencing were performed as previously described [22]. Briefly, DNA was extracted using the FastDNA SPIN Kit for Soil (MP Biomedicals, Carlsbad, CA, USA). The 16S rRNA gene was amplified with the U515F forward primer and U909R reverse primer [23]. Pyrosequencing was performed using the GS-FLX Titanium platform (Roche/454 Life Sciences, Branford, CT, USA) at the Roy J. Carver Biotechnology Center at the University of Illinois at Urbana-Champaign (IL, USA).

### Pyrosequence data analysis

Raw 16S rRNA gene sequences were screened and trimmed with QIIME 1.8.0 [24] using a sequence length (≥150 nt) and quality score (≥25) cut-off. The trimmed sequence data was clustered with the UCLUST algorithm using ≥97% sequence identity cut-off [25]. Representative sequences of each OTU were aligned using PyNAST [26] and chimeric sequences were removed using ChimeraSlayer [27]. The phylogenetic assignment of each OTU was carried out with a dataset obtained from Greengenes website (gg_13_5_otus; http://greengenes.secondgenome.com/) [28]. The Chao1 index and rarefaction curve were calculated by EstimateS (version 9.1.0) [29]. The coverage values were calculated using equation [1 – (*n* / *N*)], where *n* is the number of OTUs in a single read (singleton) and *N* is the total number of reads analyzed [30]. The weighted UniFrac distances were used for principal coordinate analysis (PCoA) [31]. Phylogenetic trees for 16S rRNA gene pytotags and previously reported sequences were constructed with the ARB program based on the neighbor-joining algorithm [32]. Insertion of pyrotag sequences (ca. 370 bp) was performed with the parsimony insertion tool of the ARB program. The topology of the trees was estimated by 1,000 bootstrap replicates [33].

### Statistical analysis

In order to correlate microbial community profiles with reactor operational conditions (ORL, HRT and reactor type), statistical analysis including redundancy analysis (RDA) and correspondence analysis (CA) were performed using CANOCO software version 4.5 (Microcomputer Power, Ithaca, NY, USA) [34]. According to the instruction of CANOCO, when the longest length lies between 3 and 4, it is reasonable to apply either linear method (RDA) or unimodal method (CA). All OTUs were used for calculation and major groups were picked out manually and plotted with operation conditions.

### Nucleotide sequences accession number

The pyrosequence data obtained in this study have been deposited under DDBJ/EMBL/GenBank accession no. DRA002423.

## Results and Discussion

### Reactor operation

The operational performance of anaerobic packed-bed (AP) and hybrid packed-bed (HP) reactors treating synthetic soft drink wastewater is shown in Fig 2 and Table 1. AP and HP were continuously operated for more than 800 days. The removal efficiency of COD consistently maintained at 93–97% with an effluent COD mostly below 100 mg L^−1^ after 77 and 12 days of operation of AP and HP, respectively. After the days of operation, no apparent differences in performance were observed between AP and HP. The total volume of methane increased gradually with an increase in OLR (Fig. 2). The average values of pH were stable during the operation, implying no obvious acid accumulation in the reactors. These results indicated that enriched microbial consortia in AP and HP retain the stability against the feeding of synthetic soft drink wastewater at 2.0 g-COD L^−1^ d^−1^. Dark gray-black-colored biofilm was formed on the surface of ceramic media in both reactors. The biofilm samples were retrieved and used for microbial community analysis (Fig. 2).

**Table 1:** Operational parameter of anaerobic packed-bed (AP) and hybrid packed-bed (HP) reactors.

Parameters	AP						HP				
Day	0–11	12–76	77–90	91–114	115–654	655–743	744–810	0–11	12–30	31–654	655–743	744–810
HRT (d)	Batch	6	5	4	3	2	1.5	Batch	6	3	2	1.5
OLR (g-COD L^−1^ d^−1^)	Batch	0.5	0.6	0.75	1.0	1.5	2.0	Batch	0.5	1.0	1.5	2.0
COD removal (%)	22.4±5.5	41.3±27.5	95.9±0.6	94.5±1.6	97.4±2.3	97.2±1.6	96.4±1.0	66.0±19.7	93.5±0.9	95.1±5.0	96.2±1.4	97.2±0.3
Methane (mL d^−1^)	N.D.	N.D.	514.4±32.5	793.6±53.9	1216.1±242.8	2657.1±424.6	3642.2±218.4	N.D.	N.D.	1402.5±237.3	2478.1±391.7	3352.2±260.2
pH	7.3±0.3	7.4±0.1	7.4±0.2	7.5±0.1	7.3±0.3	7.0±0.1	7.1±0.1	7.6±0.2	7.5±0.2	7.4±0.3	7.1±0.1	7.1±0.1

AP, anaerobic packed-bed reactor; HP, hybrid packed-bed reactor; COD: chemical oxygen demand; HRT, hydraulic retention time; OLR, organic loading rate; N.D. not determined.

### Overview of 16S rRNA gene pyrosequencing

**Fig 3:**
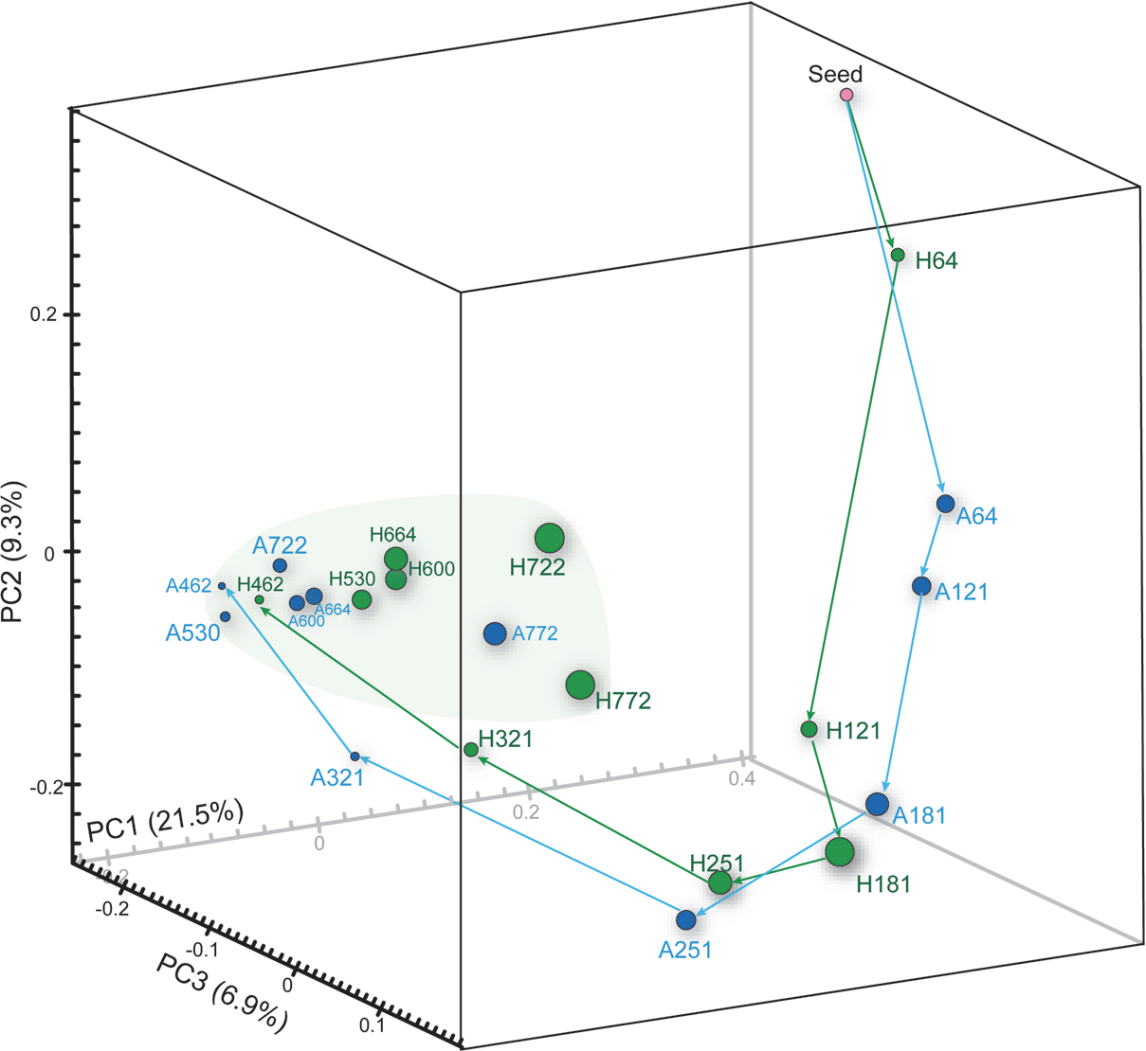
Principal coordinate analysis based on the abundances of 16S rRNA gene OTUs (unweighted UniFrac). For this analysis, observed 16S rRNA gene OTUs were normalized to 1,400 reads per sample. A and H indicate the samples taken from the anaerobic packed-bed (AP) and hybrid packed-bed (HP) reactors. The numbers following A and H indicate days of the operation for biomass sampling.

**Fig 4:**
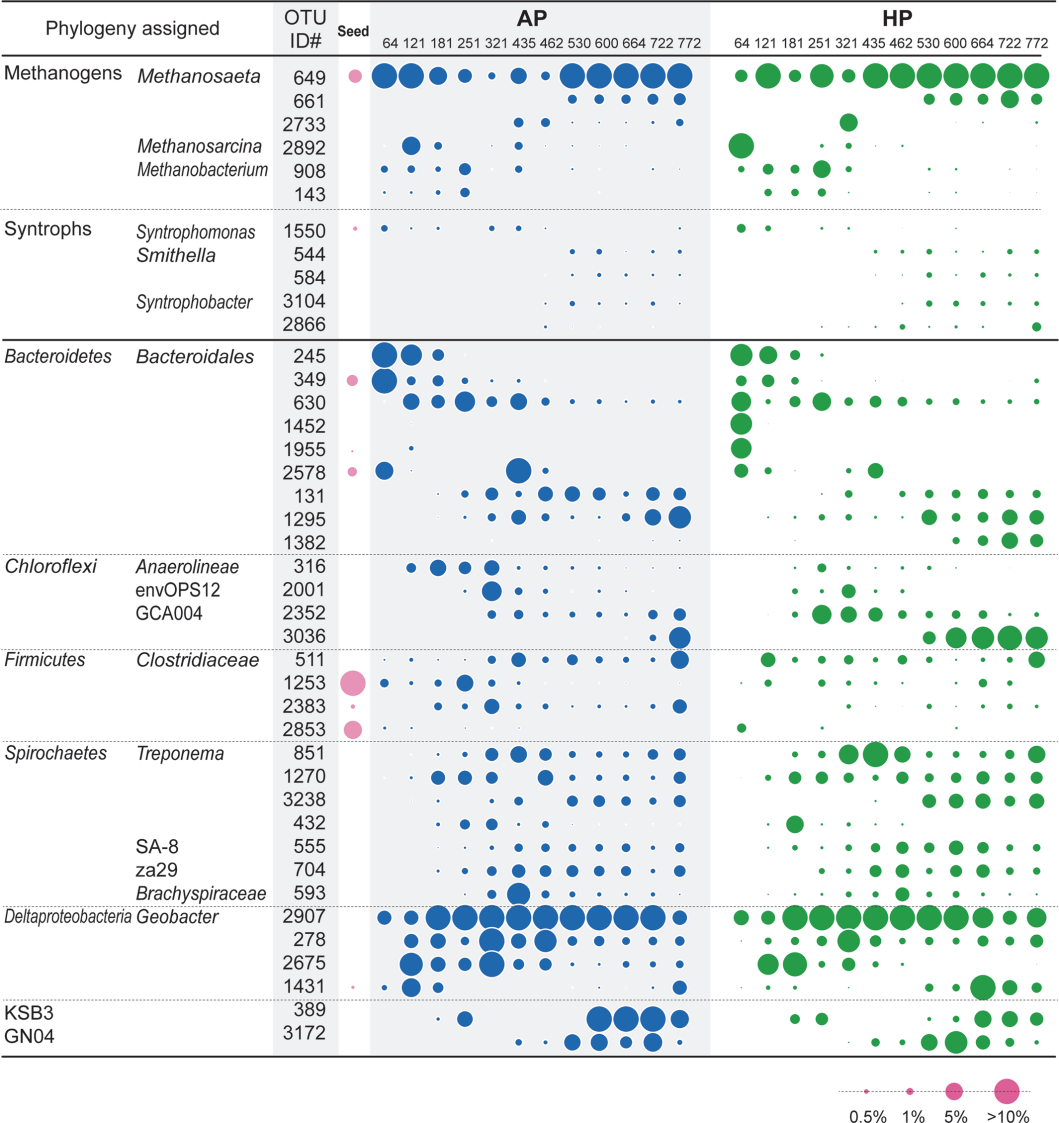
Bubble plot indicates the relative abundances of major OTUs retrieved from anaerobic packed-bed (AP) and hybrid packed-bed (HP) reactors. The numbers below AP and HP on top row indicate days of the operation for biomass sampling.

16S rRNA gene pyrotag libraries were constructed for twelve AP and HP biofilm samples each and their seed sludge. A total of 98,057 16S rRNA gene pyrotag reads were retrieved and further classified into 2,882 OTUs using a 97% sequence identity cut-off (S1 Table). Although the rarefaction curves of most samples were insufficient to achieve the plateau (S1 Fig.), the high Good’s coverage values (>93%) suggested that obtained OTUs adequately estimated the microbial diversity of the reactors. According to the Chao1 indexes, the biofilm may contain approximately 1.53–2.23-fold more OTUs than detected. Comparing microbial community composition between samples, unweighted UniFrac-based principal coordinate analysis (PCoA) clearly showed that the community composition varied with time (Fig. 3). Specifically, the microbial constituents continuously change over 321 days and reached stable structure only after 462 days, based on Jackknife clustering analysis, weighted UniFrac-based PCoA and correspondence analysis (CA) (S2, S3, and S4 Figs.). Despite the dynamic community structure, the steady COD removal indicates that the enriched microbial consortia at all stages were suitable for soft drink wastewater treatment at the respective operation conditions (Fig. 2). Using OTU-level phylogenetic analyses, we identify dominant organisms (Fig. 4) and discuss their potential ecological roles below.

### *Bacteroidetes*, *Chloroflexi*, *Firmicutes*, and *Spirochaetes*

Phyla thought to take part in the anaerobic digestion nexus [35–39], *Bacteroidetes*, *Chloroflexi*, *Firmicutes*, and *Spirochaetes*, were detected in all samples (S2 Table and Fig. 4). *Firmicutes* family *Clostridiaceae* (OTU1253, 2383, and 2853) were found in seed sludge and consistently observed throughout operation. On the other hand, although only two abundant OTUs (349 and 2758) were found in seed sludge within the phylum *Bacteroidetes*, other seven major OTUs emerged during the operation and their abundances behaved differently over time: OTUs 245, 349, 630, 1452, and 1955 predominated before day 435 and decreased in the later stages while OTUs 131, 1295, and 1382 increased after 321 days. Despite no dominant *Chloroflexi*-related OTUs in seed sludge, the abundances of three *Chloroflexi*-type OTUs (OTU316, 2001, and 2352) were frequently detected at day 121–435 and decreased after day 530, while OTU3036 predominated in later stage. Based on redundancy analysis (RDA) to correlate the abundance of major OTUs with operational conditions (S5A Fig.), HRT, OLR, and reactor type were the major explanatory variables; further, this RDA plot supported the fluctuation of the discussed *Bacteroidetes*, *Chloroflexi*, and *Firmicutes* OTUs (S5B Fig.). The members of the phyla may be responsible for fermentative degradation of protein and, more importantly, sugar to VFAs, based on previous reports [40–42]. In addition, *Bacteroidetes* found in the reactor may perform PEG degradation as a *Bacteroidetes* member, *Bacteroides* sp. PG1, has been observed to degrade PEG1000 axenically or in co-culture with *Methanobacterium* sp. DG1 [43]. While *Spirochaetes* is neither known to degrade sugars nor PEG, related OTUs (555, 704, 851, 1270, and 3238) were consistently observed after 121 days (Fig. 4 and S5B Fig.), indicating that relatively high OLR condition (>1.0 g-COD L^−1^ d^−1^) facilitated their proliferation in the reactors. Although studies have reported *Spirochaetes* populations performing syntrophic acetate oxidation [44] and acetogenesis [45] in methanogenic environments, their ecological function still remains unclear.

### Candidate phyla KSB3 and GN04

**Fig 5:**
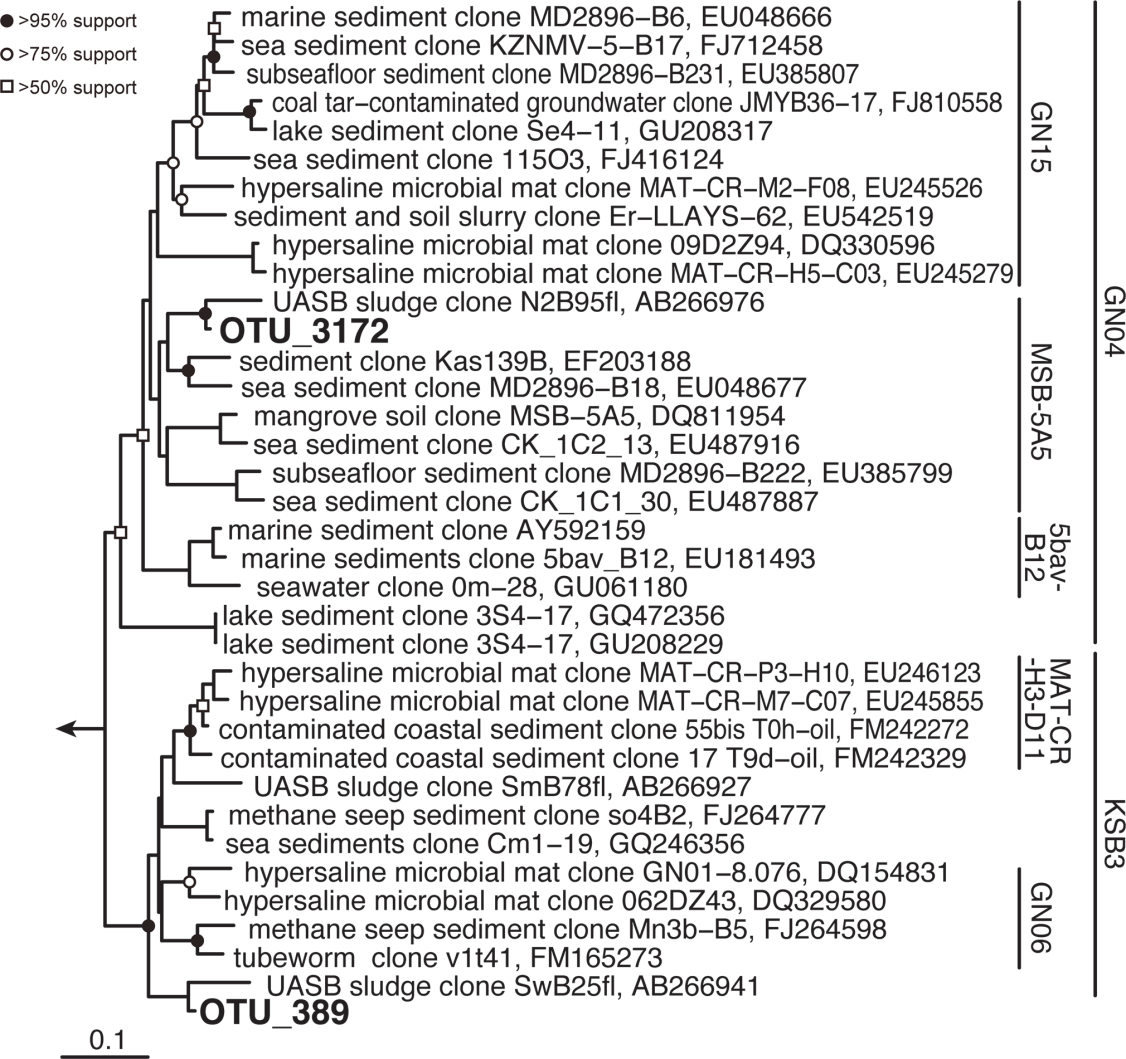
Distance matrix tree of 16S rRNA gene sequences assigned to the candidate phyla GN04 and KSB3 retrieved from anaerobic reactors based on the neighbor-joining method. Boldface indicates the sequences obtained in this study. The 16S rRNA gene sequences of *Methanosaeta harundinacea* 8Ac (AY817738), *Methanosaeta pelagica* 03d30q (AB679167), *Methanosaeta concilii* opfikon (X51423) were used as outgroup. The bar indicates 10% base substitution. Branching points supported probabilities >95%, >75%, and >50% by bootstrap analyses (based on 1,000 replicates) are indicated by solid circle, open circles, and open square, respectively.

**Fig 6:**
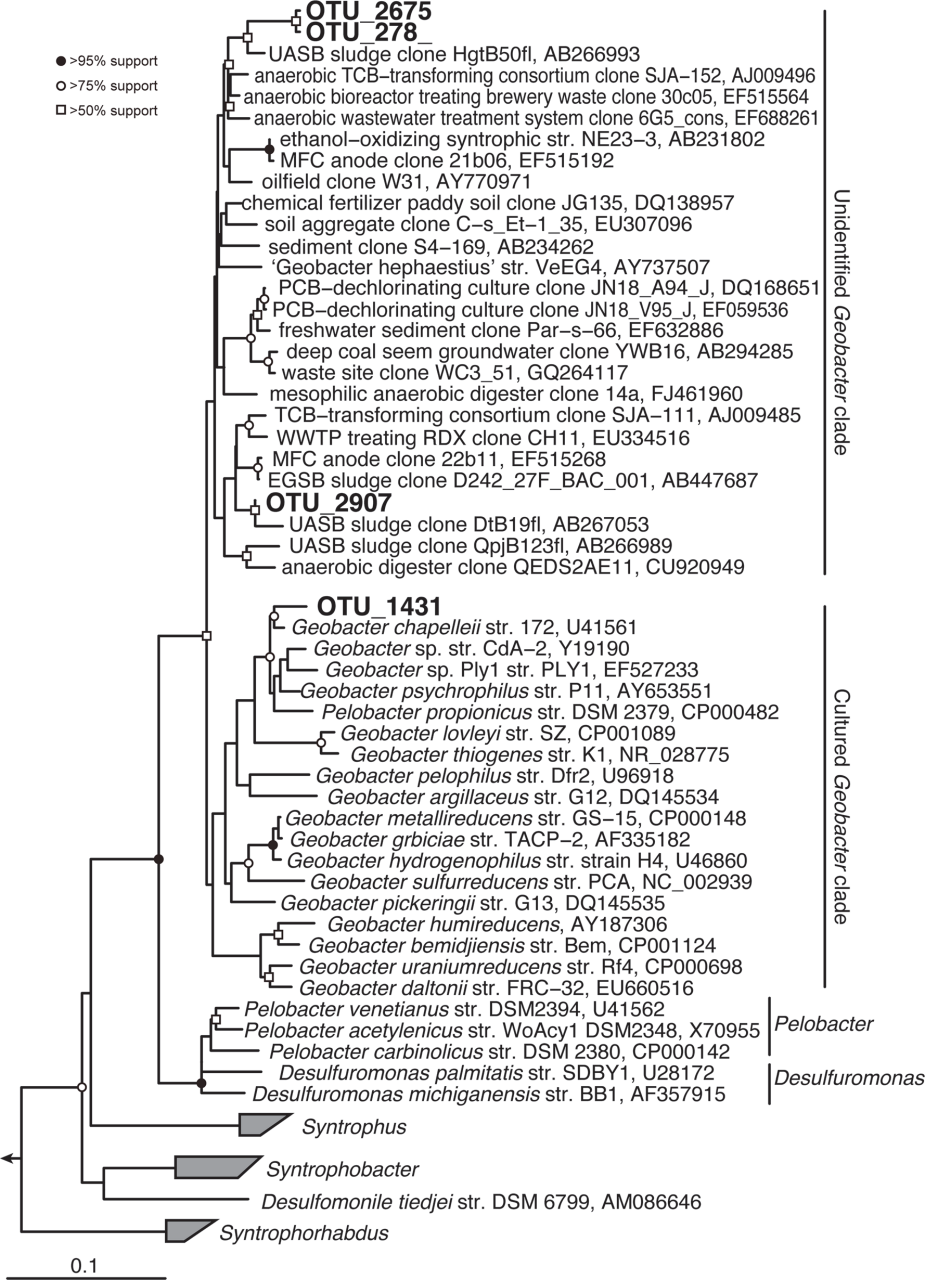
Distance matrix tree of 16S rRNA gene sequences assigned to the *Geobacter* retrieved from anaerobic reactors based on the neighbor-joining method. Boldface indicates the sequences obtained in this study. The 16S rRNA gene sequences of *Thermodesulfobacterium commune* DSM 2178 (AF418169), *Thermodesulfobacterium hveragerdense* DSM 12571 (NR_029311), and *Thermodesulfobacterium hydrogeniphilum* DSM 14290 (NR_025146) were used as outgroup. The bar indicates 10% base substitution. Branching points supported probabilities >95%, >75%, and >50% by bootstrap analyses (based on 1,000 replicates) are indicated by solid circle, open circles, and open square, respectively.

Besides such phyla widely associated with anaerobic digestion, we also observed populations of candidate phyla KSB3 and GN04 during later stages of operation (Fig. 4). After 600 days, KSB3 (OTU389) predominated up to 38.3% and 4.8% in AP and HP respectively. This KSB3 closely relates to a clone (99.2% similarity to clone SwB25fl, accession no. AB266941) associated with a mesophilic UASB reactors treating sugar-containing wastewater (Fig. 5) [35]. Further, KSB3 was also previously observed to degrade carbohydrates (i.e., glucose and maltose), especially in association with increase in influent sugar concentration [46, 47]. Thus, KSB3 likely participates in fructose degradation in both AP and HP reactors. The GN04-related OTU3172 was detected in the AP (2.6–5.6%) and HP (1.5–8.1%) reactors after 530 days operation (Fig. 4). Like KSB3, this GN04 OTU is related to a lineage (specifically MSB-5A5) associated with mesophilic UASB reactors treating sugar-containing wastewater (*e*.*g*., 99.5% identity with clone N2B95fl; accession no. AB266976) (Fig. 6) [35]. However, in both cases, their physiology and *in situ* functions remains largely unknown. The RDA plot indicated that GN04 and KSB3 populations are positioned close to the origin of the axes, indicating that their appearance could not be explained by the environmental factors tested. Further study on metagenomic and single-cell genomic analyses would provide more useful information to elucidate the ecophysiological traits of these functionally unknown microbes.

### Methanogens and syntrophs

In order to accomplish complete conversion of sugar to CH_4_ and CO_2_, it is necessary to further degrade H_2_, acetate, and other volatile fatty acids (VFAs; *e*.*g*., propionate and butyrate) likely generated from sugar fermentation by the aforementioned organisms. Specific methanogen clades are known to individually degrade H_2_ and acetate to CH_4_ and CO_2_. On the other hand, degradation of VFAs is thermodynamically limited in methanogenic environments [48–50], and syntrophs and methanogens are known to form obligate mutualistic metabolic interactions to accomplish such degradation. As expected, OTUs associated with known methanogens and syntrophs were consistently observed in AP and HP during operation (Fig. 4). For methanogens, *Methanobacterium* (OTU143 and 908) was the dominant H_2_-oxidizing methanogen throughout reactor operation. Similarly, aceticlastic *Methanosaeta*-related OTU649 was found not only in all sludge samples (1.0–27.1% of the total population) but also in seed sludge (3.1%), likely degrading acetate derived from fructose and/or PEG [51, 52]. An OTU (2892) related to *Methanosarcina*, capable of both acetate- and H_2_-oxidation, was detected at relatively higher abundances at day 121 in AP (5.5%) and day 64 in HP (12.4%). RDA revealed that *Methanosarcina-* and *Methanobacterium*-related OTUs (OTU143, 908, and 2892) were represented by relatively short arrows in the direction of HRT, indicating their proliferation at higher HRT conditions. For *Methanosaeta* populations, OTU649 had no significant correlation with HRT and OLR. In contrast, the OTU661 was strongly correlated with OLR. For *Methanosaeta* populations, OTU649 had no significant correlation with HRT and OLR. It has been reported that the affinity for acetate could be relevant to the growth of aceticlastic methanogens, and under high acetate concentrations, *Methanosarcina* spp. often outcompete *Methanosaeta* spp. [53, 54]. While the acetate concentration was not measured in the reactor, it was likely very low due to the dilution of substrate concentration from internal circulation and reactor volume right after entering the reactor. Even in such low acetate concentration, *Methanosaeta*-related OTU661 might be affected by different OLR conditions.

As for degradation of VFAs, in both reactors, we found known syntrophic populations, including *Syntrophomonas* (OTU1550), *Syntrophobacter* (OTU2866 and 3104), and *Smithella* (OTU544 and 584) (Fig. 4). Among them, *Syntrophomonas*-related OTU1550 was found in seed sludge as a major syntrophic population (0,44%). Based on characteristics of these genera [48, 55], they are most likely involved in the degradation of butyrate (*Syntrophomonas*) and propionate (*Syntrophobacter* and *Smithella*) through with syntrophic partnership with methanogens (e.g., *Methanobacterium*). Such VFAs may be produced by butyrate- or propionate-producing fermentative bacteria, such as the members of the phyla *Firmicutes* and *Bacteroidetes* [56–60]. The relatively low abundances of syntrophic bacteria (<1.6% of the total populations) are in good accordance with the results of quantitative analyses of anaerobic bioreactors with membrane hybridization [61] and sequence-specific 16S rRNA cleavage method [62]. These results suggest that hydrogenotrophic methanogens and syntrophs observed here might play a supporting role in the VFA removal to maintain process stability. RDA plot of known syntrophs showed that the OTUs associated with propionate-oxidizing syntrophs (OTU544 and 584, 2866, and 3104) shared similar trend going along with OLR axis (S5A Fig.). Given that these microbes utilize propionate as major substrate for syntrophic metabolism [48], it is reasonable to conclude that propionate fermentation might be the dominant sugar degradation pathway as OLR increased. *Syntrophomonas*-related OTU1550 that primarily utilizes butyrate, showed opposite trending with propionate oxidizers, implying a major role of butyrate fermentation in lower OLR condition.

### Geobacter

Unlike most other methanogenic environments, *Geobacter*-related organisms were frequently observed in the AP and HP reactor pyrotag libraries, although they were minor populations in seed sludge (<0.31%) (Fig. 4). OTU1431 closely related to *G*. *chapelleii* strain 172 (99.5% sequence identity; accession no. U41561), a non-fermentative, iron-reducing bacterium capable of oxidizing acetate, formate, ethanol, and lactate (Fig. 5) [63]. RDA indicated that OLR correlated with the abundance of the OTU1431 (S5A Fig.), suggesting that *G*. *chapelleii*-related organism might contribute to oxidizing acid (*i*.*e*., formate, acetate, and lactate) or alcohol (*i*.*e*., ethanol) possibly produced by fermentative degradation of sugar and PEG. Three other OTUs (278, 2675, and 2907) were distantly related to known *Geobacter* isolates (i.e., OTU278 has 98.0% identity with *G*. *argillaceus* strain G12; accession no. NR_043575, and OTU2675 and 2907 have 99.0% identity with *G*. *daltonii* strain FRC-32; accession no. NR_074916), and clustered with environmental clones that retrieved from mesophilic UASB reactors treating wastewater discharged from sugar- and amino acid-processing factories (Fig. 6) [35]. These observations suggested the importance of these *Geobacter*-related organisms in anaerobic processes treating food-processing wastewater. Within this poorly characterized *Geobacter* clade, 16S rRNA gene sequence of a syntrophic ethanol-oxidizing bacterium NE23-3 (accession no. AB231802) was deposited. Albeit no report on its physiology has yet been published, such unidentified *Geobacter* may oxidize ethanol (and possibly other syntrophic substrates) in association with hydrogenotrophic methanogens. RDA plot showed that these OTUs had no correlations with OLR/HRT. It is puzzling that *Geobacter* predominated the reactor community despite no substantial addition of oxidized metals (*e*.*g*., Fe^3+^ and Mn^4+^). However, recent studies suggest that *Geobacter* may thrive under methanogenic conditions through interspecies electron transfer with methanogens [64, 65]. In short, while we suspect they ought play an important role in the treatment of soft drink wastewater based on their consistent presence, more studies are necessary to investigate their ecological contribution.

### Conclusions

We successfully operated AP and HP reactors to treat synthetic soft drink wastewater. Based on the 16S rRNA gene pyrotag analyses, we identified core microbial constituents and assigned their possible function based on previously known physiological characteristics: *Methanosaeta*, *Methanosarcia*, and *Methanobacterium* as major methanogenic archaea; *Bacteroidetes*, *Chloroflexi*, *Firmicutes*, and KSB3 as fermentative bacteria; *Bacteroidetes* as PEG degrader. Syntrophs, *Syntrophomonas*, *Syntrophobacter*, and *Smithella* may support degradation of VFAs derived from sugar and PEG degradation by the fermenters. While we also identify *Geobacter*, *Spirochaetes*, and GN04 members prevalent in the reactor, their ecological role in soft drink wastewater treatment remains unclear. Interestingly, many of these organisms, especially KSB3 and GN04, appear to be strongly influenced by operational conditions, indicating that specific organisms may be adapted to and responsible for sugar/PEG degradation under specific conditions.

## Supporting Information

S1 Fig Rarefaction curves of 16S rRNA gene sequences of (A) anaerobic packed-bed (AP) and (B) hybrid packed-bed (HP) reactors. (TIF)

S2 Fig Jackknife clustering of 16S rRNA gene pyrotag libraries from anaerobic packed-bed (AP) and hybrid packed-bed (HP) reactors based on (A) unweighted and (B) weighted UniFrac normalized to 1,400 reads per sample. “Cluster” indicates the grouped samples showed in Fig. 3 (unweighted) and S3 Fig. (weighted).
(TIF)

S3 Fig Principal coordinate analysis (PCoA) based on the abundances of 16S rRNA gene OTUs (weighted UniFrac). For this analysis, observed 16S rRNA gene OTUs were normalized to 1,400 reads per sample. A and H indicate the samples taken from the anaerobic packed-bed (AP) and hybrid packed-bed (HP) reactors. The numbers following A and H indicate days of the operation for biomass sampling.
(TIF)

S4 Fig Correspondence analysis (CA) based on the abundances of 16S rRNA gene OTUs. A and H indicate the samples taken from the anaerobic packed-bed (AP) and hybrid packed-bed (HP) reactors. The numbers following A and H indicate days of the operation for biomass sampling.
(TIF)

S5 Fig Redundancy analysis (RDA) based on the abundances of 16S rRNA gene OTUs of (A) known methanogens, syntrophs and *Geobacter* populations and (B) the phyla *Bacteroidetes*, *Chloroflexi*, *Firmicutes*, and *Spirochaetes* and candidate phyla KSB3 and GN04 populations. (TIF)

S1 Table Pyrosequencing results of 16S rRNA genes amplicon reads from anaerobic packed-bed (AP) and hybrid packed-bed (HP) reactors. (PDF)

S2 Table Microbial community composition of anaerobic packed-bed (AP) and hybrid packed-bed (HP) reactors. (PDF)
