# Supplementary figures and images for: Microbial Community Analysis of Anaerobic Reactors Treating Soft Drink Wastewater
Source: PLoS One. 2015 Mar 6;10(3):e0119131. doi: 10.1371/journal.pone.0119131 (PMC4352018; doi:10.1371/journal.pone.0119131)

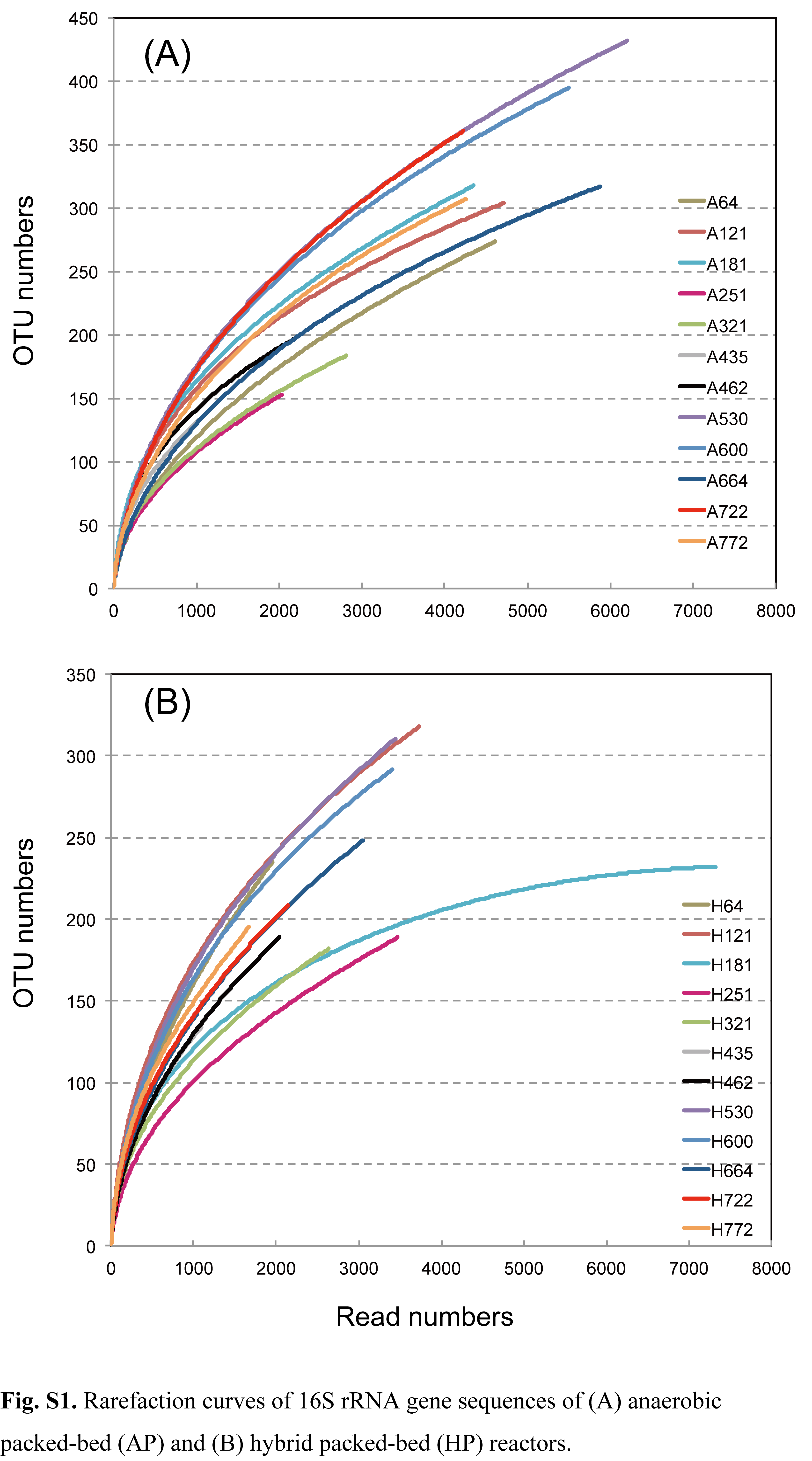

Supplement: S1 Fig — (TIF) [file pone.0119131.s001.tif]

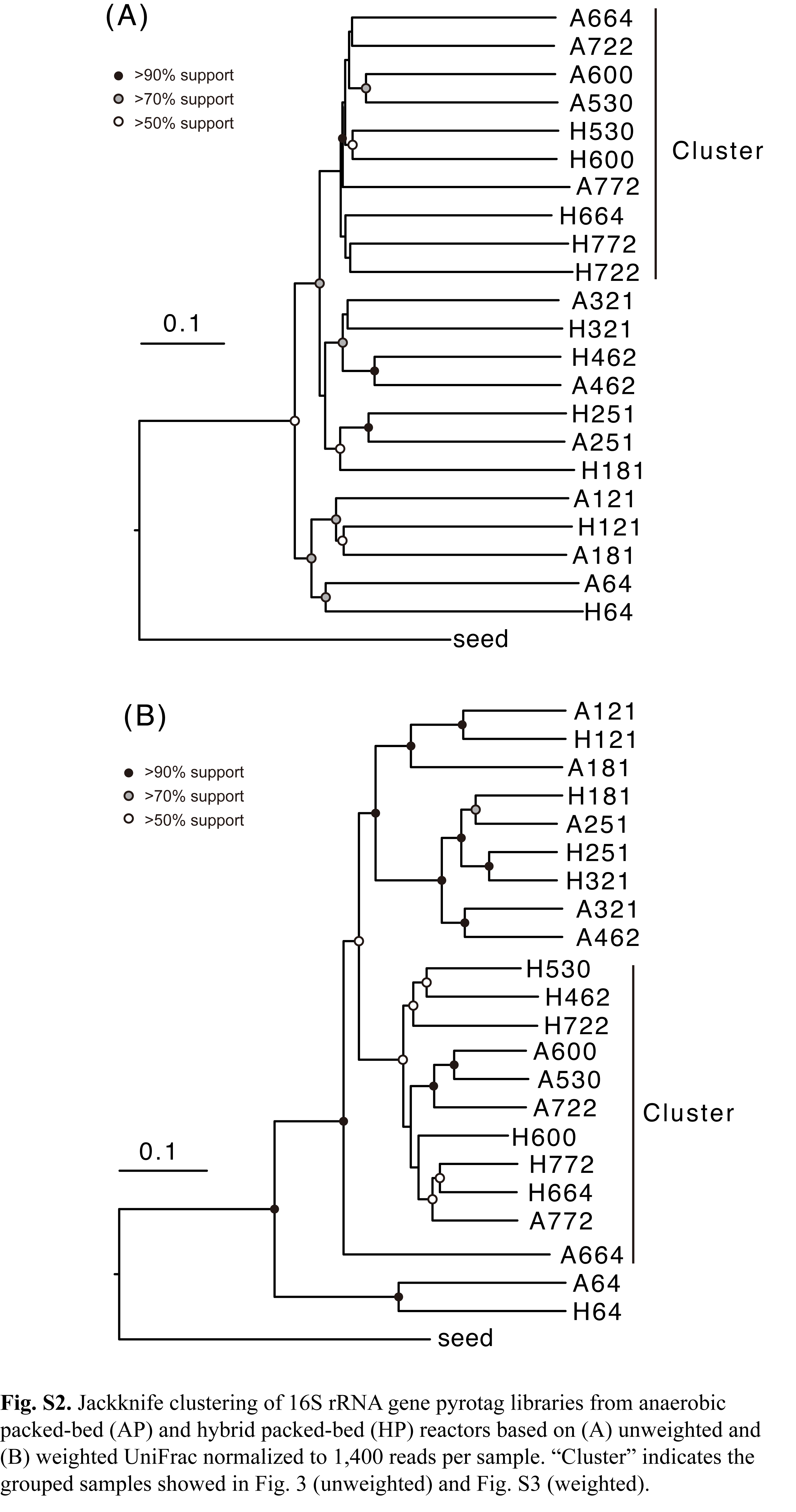

Supplement: S2 Fig — “Cluster” indicates the grouped samples showed in Fig. 3 (unweighted) and S3 Fig. (weighted). (TIF) [file pone.0119131.s002.tif]

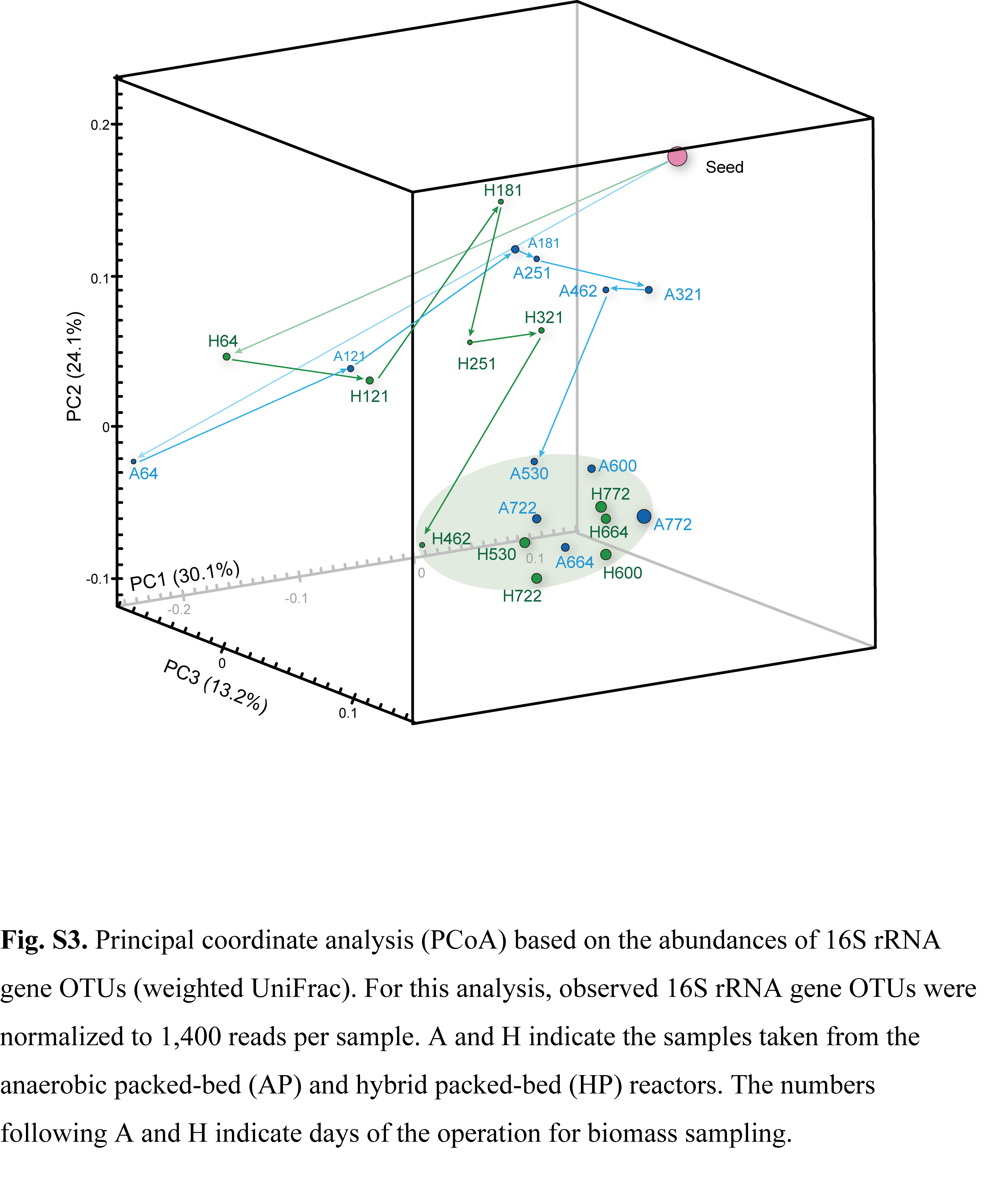

Supplement: S3 Fig — For this analysis, observed 16S rRNA gene OTUs were normalized to 1,400 reads per sample. A and H indicate the samples taken from the anaerobic packed-bed (AP) and hybrid packed-bed (HP) reactors. The numbers following A and H indicate days of the operation for biomass sampling. (TIF) [file pone.0119131.s003.tif]

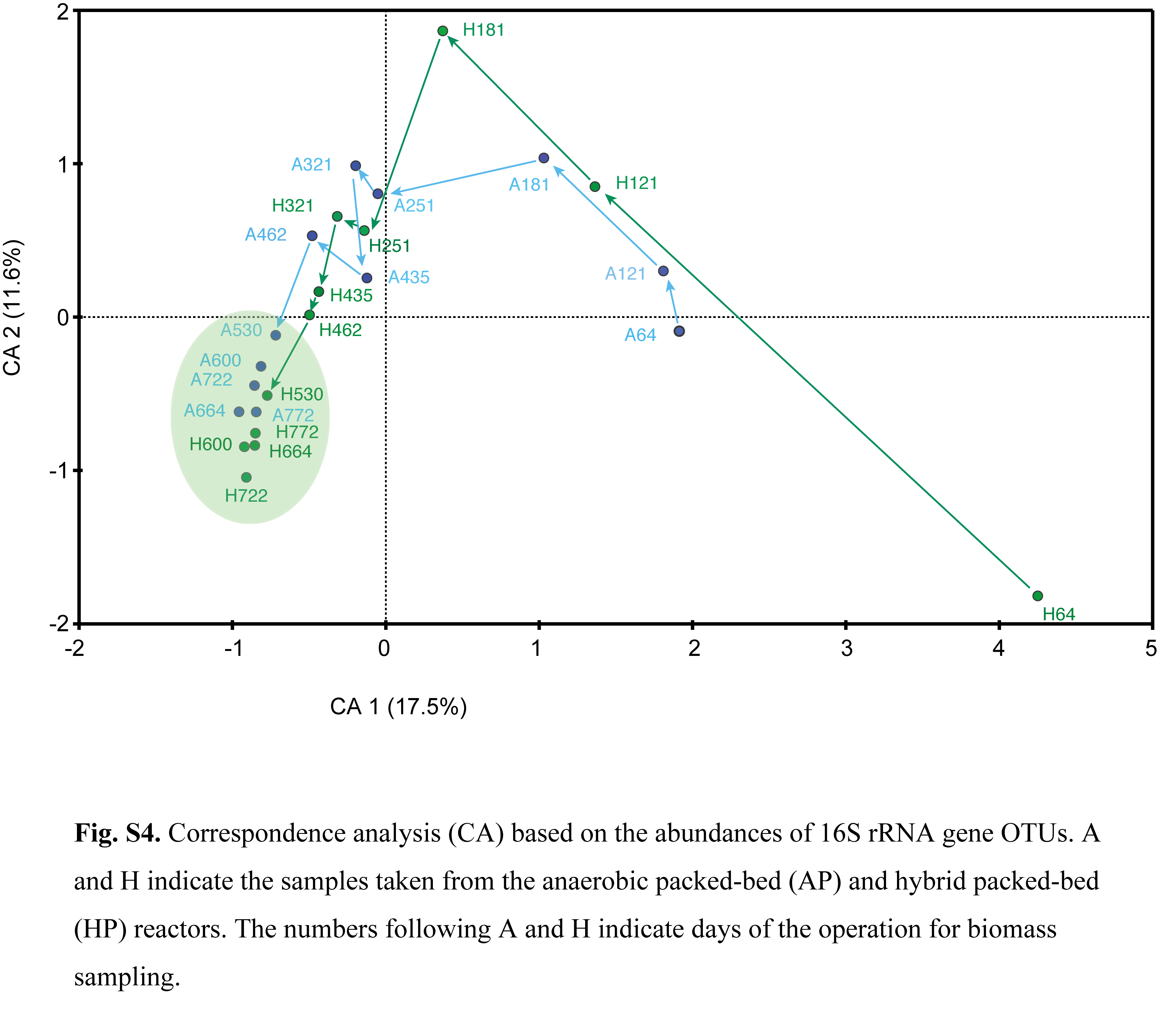

Supplement: S4 Fig — A and H indicate the samples taken from the anaerobic packed-bed (AP) and hybrid packed-bed (HP) reactors. The numbers following A and H indicate days of the operation for biomass sampling. (TIF) [file pone.0119131.s004.tif]

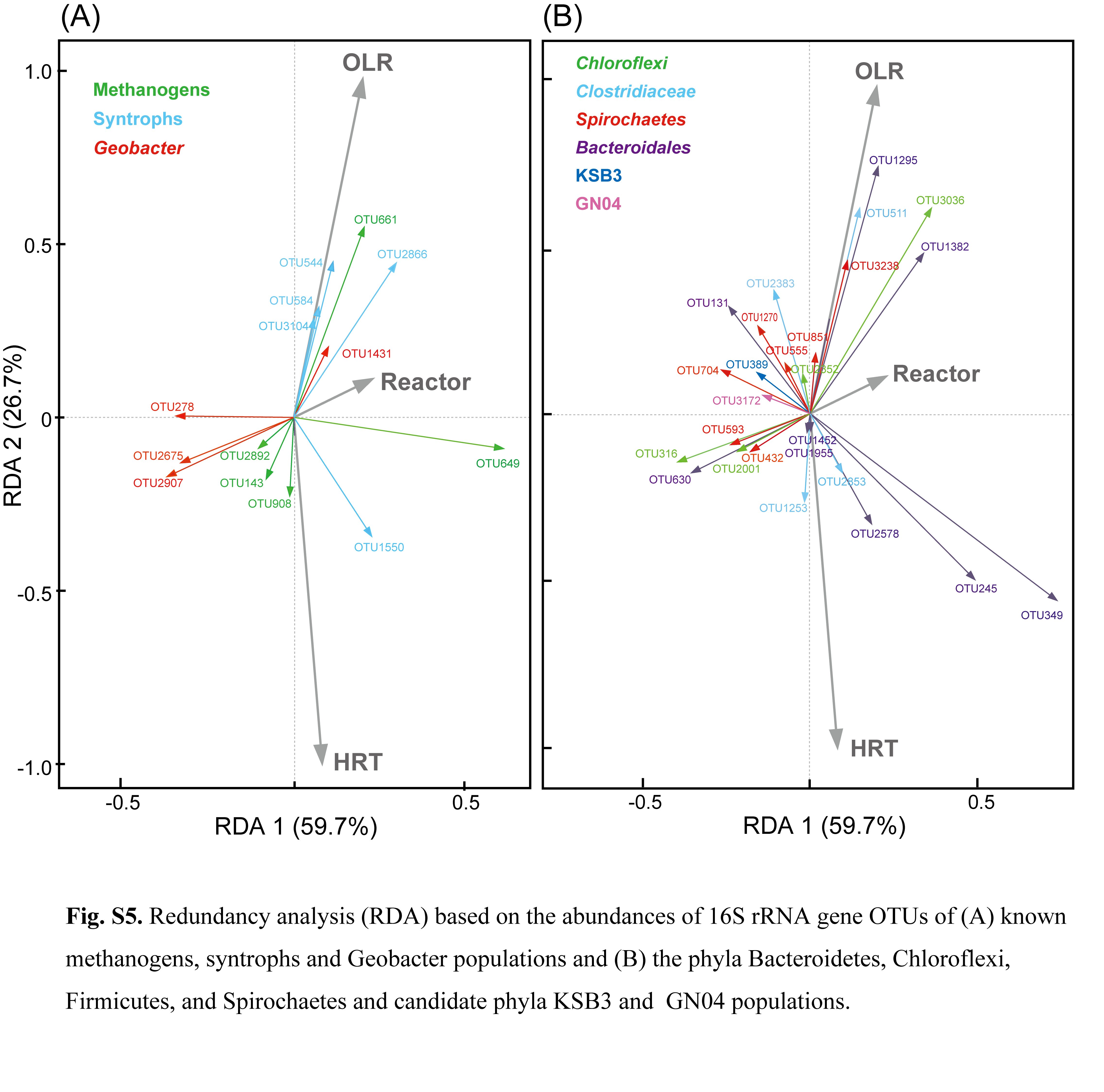

Supplement: S5 Fig — (TIF) [file pone.0119131.s005.tif]
