# Supplementary material for: Microbial Community Analysis of Anaerobic Reactors Treating Soft Drink Wastewater
Source: PLoS One. 2015 Mar 6;10(3):e0119131. doi: 10.1371/journal.pone.0119131 (PMC4352018; doi:10.1371/journal.pone.0119131)
Supplement: S1 Table — (PDF) [file pone.0119131.s006.pdf]

Table S1. Pyrosequencing results of 16S rRNA genes amplicon reads from anaerobic packed-bed (AP) and hybrid packed-bed (HP) reactors.

	seed	Operation days at sampling on AP reactor												Operation days at sampling on HP reactor											
		64	121	181	251	321	435	462	530	600	664	722	772	64	121	181	251	321	435	462	530	600	664	722	772
Total 16S pyrotag reads	14,090	4,622	4,717	4,363	2,035	2,823	1,031	2,213	6,215	5,510	5,881	4,229	4,262	1,955	3,737	7,336	3,478	2,637	1,105	2,045	3,458	3,415	3,069	2,147	1,684
Total OTU number (>97% identity)	1,008	274	304	318	153	184	133	198	432	395	317	361	307	235	318	232	189	182	135	189	311	292	249	209	196
Good's coverage	96.8	96.8	97.4	96.7	96.2	96.8	93.8	96.3	96.8	96.7	97.6	95.8	96.8	93.3	96.4	99.9	97.2	96.7	93.9	95.3	95.8	96.3	96.0	95.0	93.7
Chao1	1,680	552	525	612	341	338	259	302	716	688	533	682	522	485	506	232	401	332	220	355	622	487	429	392	505

AP, anaerobic packed-bed reactor; HP, hybrid packed-bed reactor; OTU,operational taxonomic unit.
