# Supplementary material for: Microbial Community Analysis of Anaerobic Reactors Treating Soft Drink Wastewater
Source: PLoS One. 2015 Mar 6;10(3):e0119131. doi: 10.1371/journal.pone.0119131 (PMC4352018; doi:10.1371/journal.pone.0119131)
Supplement: S2 Table — (PDF) [file pone.0119131.s007.pdf]

Table S2. Microbial community composition of anaerobic packed-bed (AP) and hybrid packed-bed (HP) reactors.

| Group | Seed | Sampling date on AP reactor (population %) | | | | | | | | | | | | Sampling date on HP reactor (population %) | | | | | | | | | | | |
|---|---|---|---|---|---|---|---|---|---|---|---|---|---|---|---|---|---|---|---|---|---|---|---|---|---|
| | | 64 | 121 | 181 | 251 | 321 | 435 | 462 | 530 | 600 | 664 | 722 | 772 | 64 | 121 | 181 | 251 | 321 | 435 | 462 | 530 | 600 | 664 | 722 | 772 |
| *Bacteria* | | | | | | | | | | | | | | | | | | | | | | | | | |
| *Deltaproteobacteria* | 4.414 | 6.859 | 24.02 | 22.92 | 43.69 | 35.85 | 18.33 | 41.35 | 33.56 | 28.62 | 16.44 | 23.81 | 13.33 | 8.747 | 15.36 | 43.62 | 44.74 | 42.97 | 24.52 | 24.79 | 24.41 | 23.69 | 28.67 | 14.53 | 22.21 |
| *Bacteroidetes* | 18.23 | 43.05 | 20.9 | 14.6 | 10.61 | 8.466 | 22.41 | 8.224 | 6.404 | 5.118 | 2.925 | 11.94 | 13.75 | 40.77 | 20.93 | 13.94 | 8.309 | 7.167 | 7.783 | 4.352 | 8.473 | 6.442 | 8.048 | 12.95 | 11.4 |
| *Chloroflexi* | 8.559 | 4.998 | 10.73 | 11.96 | 10.66 | 19.45 | 9.602 | 10.8 | 9.22 | 7.042 | 3.877 | 6.172 | 13.42 | 3.734 | 10.2 | 7.266 | 11.53 | 12.51 | 18.46 | 11.2 | 10.29 | 12.39 | 12.41 | 12.76 | 10.93 |
| *Firmicutes* | 35.64 | 9.022 | 12.51 | 15.4 | 9.73 | 11.19 | 9.214 | 5.92 | 4.94 | 3.466 | 2.398 | 3.452 | 12.79 | 16.57 | 12.95 | 8.451 | 6.613 | 7.395 | 3.891 | 4.597 | 5.09 | 3.572 | 5.213 | 3.866 | 8.67 |
| *Spirochaetes* | 5.813 | 0.563 | 2.777 | 7.128 | 7.371 | 13.57 | 20.56 | 14.87 | 11.81 | 10.85 | 9.046 | 7.756 | 15.79 | 1.637 | 3.452 | 11.21 | 5.837 | 11.6 | 20.63 | 17.8 | 11.65 | 16.31 | 16.45 | 13.97 | 20.84 |
| *Nitrospirae* | 0.035 | 0.022 | 0 | 0.527 | 1.818 | 2.657 | 1.94 | 2.666 | 3.878 | 2.486 | 0.986 | 2.152 | 1.76 | 0 | 0.027 | 1.745 | 1.006 | 1.1 | 1.991 | 0.88 | 1.591 | 1.376 | 2.118 | 1.444 | 2.019 |
| *Planctomycetes* | 0.617 | 0.238 | 0.254 | 0.206 | 0.442 | 0.673 | 0.582 | 2.892 | 1.175 | 1.525 | 1.19 | 1.371 | 0.422 | 0.358 | 0.348 | 0.191 | 0.345 | 0.303 | 0.905 | 1.125 | 0.521 | 0.439 | 0.326 | 0.373 | 0.178 |
| *Chlorobi* | 0.149 | 0.26 | 0.869 | 2.75 | 1.622 | 2.373 | 1.649 | 3.163 | 4.505 | 3.448 | 2.568 | 3.358 | 1.877 | 0.051 | 0.589 | 0.981 | 0.489 | 1.441 | 0.543 | 0.636 | 3.326 | 1.083 | 0.554 | 1.025 | 0.416 |
| *Acidobacteria* | 0.085 | 0.065 | 0 | 0.115 | 0.147 | 0.638 | 0.873 | 0.587 | 0.37 | 0.599 | 0.187 | 0.851 | 0.235 | 0.051 | 0.321 | 0.191 | 0.086 | 0.379 | 1.538 | 0.538 | 0.376 | 0.264 | 0.228 | 0.326 | 0.534 |
| *Alphaproteobacteria* | 2.91 | 0.238 | 0.403 | 0.481 | 0.049 | 0.283 | 0.679 | 0.678 | 0.209 | 0.29 | 0.051 | 0.118 | 1.079 | 0.307 | 0.99 | 0.136 | 0.173 | 0.417 | 0.452 | 0.147 | 0.058 | 0.205 | 0.033 | 0.279 | 0 |
| *Caldiserica* | 0.177 | 0 | 0 | 0.138 | 0 | 0 | 0 | 0 | 0.016 | 0 | 0.068 | 0.024 | 0.023 | 0.153 | 0 | 0.055 | 0 | 0.038 | 0 | 0 | 0.058 | 0.029 | 0.065 | 0 | 0 |
| *Verrucomicrobia* | 1.065 | 0.043 | 0.085 | 0.963 | 0.049 | 0.071 | 0 | 0.09 | 0.209 | 0.109 | 0.119 | 0.071 | 0.141 | 0.205 | 0.027 | 0.136 | 0.173 | 0 | 0 | 0.049 | 0.231 | 0.146 | 0.033 | 0 | 0.059 |
| *Cyanobacteria* | 0.142 | 0.022 | 0.912 | 3.507 | 0.098 | 0.142 | 1.746 | 1.582 | 0.145 | 0.073 | 0.051 | 0.236 | 0.023 | 0 | 3.265 | 1.036 | 0.23 | 0.834 | 0.271 | 0.147 | 0.058 | 0.117 | 0.033 | 0.419 | 0.059 |
| *Armatimonadetes* | 0.028 | 0.022 | 0.127 | 0.115 | 0.098 | 0.142 | 0.097 | 0.045 | 0.241 | 0.073 | 0.034 | 0.095 | 0.047 | 0.307 | 0.134 | 0 | 0.201 | 0.076 | 0 | 0.049 | 0.087 | 0.029 | 0 | 0 | 0 |
| *Gemmatimonadetes* | 0 | 0 | 0.021 | 0.023 | 0 | 0 | 0.194 | 0.362 | 0.097 | 0.2 | 0.119 | 0.213 | 0.07 | 0 | 0.054 | 0.109 | 0.029 | 0.038 | 0.09 | 0.44 | 0.116 | 0.029 | 0 | 0.047 | 0 |
| *Betaproteobacteria* | 3.577 | 0.736 | 0.085 | 0.252 | 0 | 0.142 | 0.097 | 0.271 | 0.048 | 0.054 | 0.034 | 0.473 | 0.399 | 0.46 | 0.054 | 1.104 | 0.058 | 0.114 | 0.09 | 0.196 | 0 | 0.029 | 0.033 | 3.586 | 0.059 |
| *Actinobacteria* | 3.172 | 0.087 | 0.212 | 0.092 | 0.147 | 0 | 0 | 0 | 0.016 | 0.127 | 0 | 0 | 0.023 | 0.205 | 0.161 | 0 | 0.029 | 0 | 0 | 0 | 0.029 | 0.029 | 0 | 0.047 | 0.119 |
| *Synergistetes* | 2.413 | 0.606 | 1.23 | 0.206 | 0.049 | 0 | 0 | 0 | 0.032 | 0 | 0 | 0 | 0.094 | 3.018 | 0.428 | 0 | 0.029 | 0 | 0 | 0 | 0 | 0 | 0 | 0 | 0 |
| *Gammaproteobacteria* | 0.894 | 0.065 | 0.17 | 0 | 0 | 0.425 | 0.097 | 0.407 | 0.209 | 0.054 | 0.051 | 0.047 | 0.188 | 0.205 | 0.054 | 0.041 | 0.086 | 0.265 | 0.633 | 0.147 | 0.087 | 0.029 | 0.033 | 0.419 | 0.178 |
| *Thermotogae* | 0.61 | 0.087 | 0 | 0.023 | 0 | 0 | 0 | 0 | 0 | 0 | 0 | 0 | 0 | 0 | 0.348 | 0.027 | 0 | 0 | 0 | 0 | 0 | 0 | 0 | 0 | 0 |
| *Tenericutes* | 0.426 | 0.173 | 0.721 | 0.367 | 0 | 0 | 0 | 0 | 0 | 0 | 0 | 0 | 0 | 0.102 | 0.455 | 0.191 | 0 | 0.038 | 0 | 0 | 0 | 0 | 0 | 0 | 0 |
| *Fusobacteria* | 0.177 | 0 | 0 | 0.069 | 0 | 0.071 | 0 | 0 | 0 | 0 | 0 | 0 | 0 | 0 | 0 | 0 | 0 | 0 | 0 | 0 | 0 | 0 | 0 | 0 | 0 |
| *Fibrobacteres* | 0.106 | 0 | 0 | 0 | 0 | 0 | 0 | 0 | 0 | 0 | 0 | 0 | 0 | 0 | 0 | 0 | 0 | 0 | 0 | 0 | 0 | 0 | 0 | 0 | 0 |
| *Lentisphaerae* | 0.099 | 0.13 | 0 | 0.023 | 0 | 0 | 0 | 0 | 0 | 0 | 0 | 0 | 0 | 0.051 | 0 | 0 | 0 | 0.038 | 0 | 0 | 0 | 0 | 0 | 0 | 0 |
| *Epsilonproteobacteria* | 0.192 | 0 | 0.021 | 0 | 0 | 0 | 0 | 0 | 0 | 0.018 | 0 | 0 | 0 | 0.051 | 0 | 0 | 0 | 0.038 | 0 | 0 | 0 | 0 | 0 | 0 | 0.059 |
| *Chlamydiae* | 0.035 | 0 | 0 | 0.115 | 0.197 | 0.035 | 0.194 | 0.136 | 0.177 | 0.109 | 0.153 | 0.047 | 0.399 | 0 | 0.294 | 0.082 | 0.029 | 0.265 | 0 | 0.098 | 0.231 | 0.088 | 0.065 | 0.14 | 0.416 |
| *Elusimicrobia* | 0.014 | 0.022 | 0.085 | 0 | 0 | 0 | 0.097 | 0.045 | 0.016 | 0.054 | 0 | 0.024 | 0 | 0.051 | 0.054 | 0 | 0 | 0 | 0 | 0 | 0 | 0 | 0 | 0 | 0 |
| *Thermi* | 0 | 0 | 0 | 0 | 0 | 0 | 0.097 | 0 | 0 | 0 | 0 | 0 | 0 | 0 | 0 | 0 | 0 | 0 | 0 | 0 | 0 | 0 | 0 | 0 | 0 |
| | | | | | | | | | | | | | | | | | | | | | | | | | |
| Candidate phyla | | | | | | | | | | | | | | | | | | | | | | | | | |
| GN04 | 0 | 0 | 0 | 0 | 0 | 0 | 0.873 | 0.452 | 4.65 | 4.973 | 2.67 | 6.148 | 0.587 | 0 | 0 | 0 | 0 | 0.152 | 1.357 | 1.125 | 5.35 | 11.98 | 3.845 | 1.723 | 0.713 |
| KSB3 | 0 | 0 | 0 | 0.321 | 4.029 | 0 | 0 | 0 | 0 | 12.2 | 38.63 | 12.49 | 5.443 | 0 | 0 | 1.772 | 2.703 | 0 | 0 | 0 | 0.405 | 0.966 | 5.018 | 4.285 | 3.088 |
| FCPU426 | 0.057 | 0 | 0 | 0 | 0 | 0 | 0 | 0 | 0.354 | 1.978 | 0.595 | 0.189 | 0.094 | 0 | 0 | 0 | 0 | 0 | 1.81 | 4.059 | 2.776 | 0.996 | 1.075 | 0.559 | 0.178 |
| OP3 | 0.27 | 0.022 | 0.53 | 3.805 | 0.344 | 0 | 0 | 0 | 0.016 | 0 | 0 | 0.024 | 0.023 | 0 | 2.301 | 0.736 | 0.259 | 0 | 0 | 0 | 0 | 0 | 0 | 0 | 0.059 |
| WPS-2 | 0.021 | 0 | 0.064 | 0.138 | 0.197 | 1.204 | 0 | 0.09 | 0.016 | 0.036 | 0.119 | 0.095 | 0.328 | 0.051 | 0.214 | 0.027 | 0.029 | 0.341 | 0.09 | 0 | 0 | 0.029 | 0.065 | 0.186 | 0.238 |
| NKB19 | 0.376 | 0.065 | 0.042 | 0.092 | 0 | 0 | 0.097 | 0.316 | 0.064 | 0.036 | 0.068 | 0.024 | 0.047 | 0.256 | 0.08 | 0.164 | 0 | 0.038 | 0.09 | 0.489 | 0.174 | 0.059 | 0 | 0.047 | 0 |
| WSA2 | 0.298 | 0.022 | 0.127 | 0 | 0 | 0.035 | 0.097 | 0 | 0 | 0.054 | 0 | 0 | 0 | 0.409 | 0.08 | 0.027 | 0 | 0 | 0.181 | 0 | 0 | 0.059 | 0 | 0 | 0.059 |
| BRC1 | 0.085 | 0.043 | 0.064 | 0.023 | 0 | 0.035 | 0 | 0.045 | 0 | 0 | 0 | 0.189 | 0 | 0.46 | 0.134 | 0.055 | 0.058 | 0 | 0 | 0.049 | 0.029 | 0.029 | 0 | 0.047 | 0 |
| OP11 | 0.035 | 0 | 0.678 | 1.352 | 0.197 | 0.142 | 0 | 0.181 | 0.241 | 0.036 | 0.051 | 0.024 | 0.07 | 0.051 | 0.268 | 0 | 0.23 | 0.038 | 0 | 0.049 | 0.058 | 0 | 0.033 | 0 | 0 |
| WWE1 | 2.86 | 0.627 | 1.442 | 0.894 | 0 | 0.319 | 0 | 0.587 | 0 | 0 | 0 | 0 | 0 | 2.097 | 2.542 | 0.055 | 0.029 | 0 | 0 | 0.098 | 0 | 0 | 0 | 0 | 0 |
| Hyd24-12 | 0.745 | 0 | 0 | 0 | 0 | 0 | 0 | 0 | 0 | 0 | 0 | 0 | 0 | 0.051 | 0 | 0 | 0 | 0 | 0 | 0 | 0 | 0 | 0 | 0 | 0 |
| OP9 | 0.22 | 0.022 | 0 | 0 | 0 | 0.035 | 0 | 0 | 0 | 0.036 | 0 | 0 | 0 | 0.102 | 0 | 0 | 0 | 0 | 0 | 0 | 0 | 0 | 0 | 0 | 0 |
| OD1 | 0.085 | 0.043 | 0.064 | 0.527 | 0.049 | 0 | 0 | 0 | 0.209 | 0.091 | 0.51 | 0.024 | 0.375 | 0.665 | 0.562 | 0.027 | 0.345 | 0.19 | 0.09 | 0 | 0.289 | 0.117 | 0.033 | 0 | 0.653 |
| WS1 | 0.078 | 0 | 0.042 | 0 | 0 | 0 | 0 | 0 | 0 | 0.036 | 0.034 | 0 | 0 | 0 | 0.054 | 0.082 | 0.086 | 0 | 0 | 0.196 | 0 | 0 | 0 | 0 | 0 |
| OP8 | 0.064 | 0.022 | 0 | 0.023 | 0 | 0 | 0 | 0.09 | 0 | 0 | 0 | 0 | 0 | 0.102 | 0.08 | 0 | 0 | 0 | 0 | 0 | 0 | 0.088 | 0.033 | 0 | 0 |
| TM6 | 0.021 | 0.173 | 0.021 | 0.069 | 0.344 | 0.071 | 0.873 | 0.226 | 0.257 | 0.218 | 0.136 | 0.307 | 0.07 | 0 | 0.161 | 0.109 | 0.115 | 0.417 | 0.181 | 0.538 | 0.347 | 0.146 | 0.098 | 0.093 | 0.238 |
| SAR406 | 0.014 | 0 | 0 | 0 | 0 | 0 | 0 | 0 | 0 | 0 | 0 | 0 | 0 | 0 | 0 | 0 | 0 | 0 | 0 | 0 | 0 | 0 | 0 | 0 | 0 |
| GN02 | 0.014 | 0 | 0 | 0 | 0 | 0.213 | 0.097 | 0.045 | 0.257 | 0.018 | 0.017 | 0 | 0.023 | 0 | 0 | 0 | 0 | 0.038 | 0 | 0.049 | 0.087 | 0.088 | 0 | 0 | 0 |
| WS4 | 0.007 | 0 | 0 | 0 | 0 | 0 | 0 | 0 | 0 | 0 | 0 | 0 | 0 | 0 | 0 | 0 | 0 | 0 | 0 | 0 | 0 | 0 | 0 | 0 | 0 |
| WS3 | 0.007 | 0 | 0 | 0 | 0 | 0 | 0 | 0 | 0.097 | 0.054 | 0.153 | 0.307 | 0 | 0 | 0 | 0 | 0 | 0 | 0 | 0 | 0.029 | 0 | 0 | 0.047 | 0 |
| TM7 | 0.007 | 0 | 0 | 0 | 0 | 0 | 0 | 0 | 0 | 0 | 0 | 0 | 0 | 0 | 0 | 0 | 0 | 0 | 0 | 0 | 0 | 0 | 0 | 0 | 0 |
| SR1 | 0.007 | 0 | 0 | 0 | 0 | 0 | 0 | 0 | 0 | 0 | 0 | 0 | 0 | 0 | 0 | 0 | 0 | 0 | 0 | 0 | 0 | 0 | 0 | 0 | 0 |
| TA18 | 0.007 | 0 | 0 | 0 | 0 | 0 | 0 | 0 | 0 | 0 | 0 | 0 | 0 | 0 | 0 | 0 | 0 | 0 | 0 | 0 | 0 | 0 | 0 | 0 | 0 |
| NC10 | 0 | 0.022 | 0 | 0 | 0 | 0 | 0 | 0.045 | 0.145 | 0.054 | 0.034 | 0.024 | 0 | 0 | 0 | 0 | 0 | 0 | 0 | 0 | 0 | 0.059 | 0 | 0 | 0 |
| LD1 | 0 | 0 | 0 | 0.573 | 0.147 | 0.035 | 0.097 | 0 | 0 | 0 | 0 | 0 | 0 | 0 | 0.187 | 0.273 | 0.086 | 0.038 | 0.09 | 0 | 0 | 0 | 0 | 0 | 0 |
| FBP | 0 | 0 | 0 | 0 | 0 | 0 | 0 | 0 | 0 | 0 | 0 | 0 | 0 | 0 | 0.027 | 0 | 0 | 0 | 0 | 0 | 0 | 0 | 0 | 0 | 0 |
| | | | | | | | | | | | | | | | | | | | | | | | | | |
| *Archaea* | | | | | | | | | | | | | | | | | | | | | | | | | |
| *Methanosaeta* | 3.059 | 27.13 | 10.26 | 5.226 | 3.292 | 1.027 | 6.208 | 3.389 | 14.9 | 14.66 | 15.54 | 16.48 | 16.14 | 2.66 | 15.44 | 2.617 | 8.741 | 8.381 | 13.3 | 24.89 | 22.35 | 17.69 | 14.73 | 26.13 | 16.21 |
| *Methanosarcina* | 0 | 0.022 | 5.491 | 1.192 | 0 | 0.106 | 1.261 | 0.09 | 0.129 | 0.073 | 0.102 | 0 | 0 | 12.43 | 0 | 0 | 0.374 | 0.796 | 0.09 | 0.196 | 0.029 | 0 | 0 | 0 | 0.059 |
| *Methanospirillum* | 0.412 | 0.043 | 1.866 | 0.367 | 0 | 0 | 0 | 0 | 0 | 0 | 0.034 | 0 | 0 | 1.381 | 0.161 | 0 | 0 | 0 | 0 | 0 | 0 | 0 | 0 | 0 | 0 |
| *Methanolinea* | 0.646 | 0.065 | 0.021 | 0.092 | 0 | 0.035 | 0 | 0 | 0.08 | 0.091 | 0.068 | 0.118 | 0.023 | 0.205 | 0.134 | 0.055 | 0.086 | 0.038 | 0.181 | 0.196 | 0.145 | 0.176 | 0.033 | 0.14 | 0 |
| *Methanoculleus* | 0.078 | 0 | 0 | 0 | 0 | 0 | 0 | 0 | 0 | 0 | 0 | 0 | 0 | 0 | 0.027 | 0 | 0 | 0 | 0 | 0 | 0 | 0 | 0 | 0 | 0 |
| Other *Methanomicrobiales* | 0.007 | 0.043 | 0 | 0.023 | 0 | 0 | 0 | 0 | 0 | 0 | 0 | 0 | 0 | 0.153 | 0.054 | 0 | 0 | 0.038 | 0 | 0 | 0 | 0 | 0 | 0 | 0 |
| *Methanobacterium* | 0 | 1.341 | 1.378 | 1.261 | 4.226 | 0.035 | 1.067 | 0 | 0.241 | 0.145 | 0.017 | 0.213 | 0.117 | 0.972 | 3.211 | 2.672 | 6.498 | 2.162 | 0 | 0 | 0.492 | 0.439 | 0.065 | 0.186 | 0.119 |
| *Methanobrevibacter* | 0.035 | 0.519 | 0.042 | 0 | 0 | 0 | 0 | 0 | 0 | 0 | 0 | 0 | 0 | 0.256 | 0 | 0 | 0 | 0 | 0 | 0 | 0 | 0 | 0 | 0 | 0 |
| *Other Methanobacteriaceae* | 0 | 0 | 0 | 0 | 0 | 0 | 0 | 0 | 0.016 | 0.018 | 0.017 | 0 | 0 | 0 | 0 | 0 | 0 | 0 | 0 | 0 | 0.029 | 0 | 0 | 0 | 0 |
| *Methanomassiliicoccaceae* | 0.05 | 0.151 | 0.021 | 0.023 | 0.049 | 0.071 | 0.485 | 0.09 | 0.177 | 0.091 | 0.034 | 0.142 | 0.023 | 0.358 | 0.107 | 0.027 | 0.029 | 0.152 | 0 | 0.049 | 0.029 | 0 | 0.065 | 0 | 0.059 |
| *Crenarchaeota* | 0.014 | 0.043 | 0.021 | 0.023 | 0 | 0 | 0.097 | 0.045 | 0.097 | 0.054 | 0.102 | 0.118 | 0.117 | 0 | 0.054 | 0.055 | 0 | 0 | 0.181 | 0.293 | 0.231 | 0.322 | 0.098 | 0.14 | 0 |
| *Parvarchaeota* | 0.192 | 0.151 | 0 | 0 | 0 | 0 | 0 | 0 | 0 | 0 | 0 | 0 | 0 | 0 | 0 | 0 | 0 | 0 | 0 | 0 | 0 | 0 | 0 | 0 | 0 |
| | | | | | | | | | | | | | | | | | | | | | | | | | |
| Unassigned | 0.639 | 2.12 | 1.696 | 1.994 | 0.344 | 0.461 | 0.291 | 0.226 | 0.772 | 0.726 | 0.765 | 0.875 | 0.657 | 0.307 | 3.639 | 0.736 | 0.345 | 0.152 | 0.543 | 0.538 | 0.463 | 0.469 | 0.489 | 0.233 | 0.178 |
